# Site-specific SUMOylation of viral polymerase processivity factor: a way of localizingtoND10 subnuclear domains for restricted and self-controlled reproduction of herpesvirus

**DOI:** 10.1080/21505594.2021.2000689

**Published:** 2021-12-16

**Authors:** Shuyan Lai, Mengqiong Xu, Yaohao Wang, Ruilin Li, Chuan Xia, Sisi Xia, Jun Chen

**Affiliations:** a Guangdong Provincial Key Laboratory of Virology, Institute of Medical Microbiology, Jinan University, Guangzhou, China; b National Key Laboratory of Crop Genetic Improvement, College of Life Science and Technology, Huazhong Agricultural University, Wuhan, China; c Foshan Institute of Medical Microbiology, Foshan, Guangdong, China

**Keywords:** Human cytomegalovirus (HCMV), viral polymerase processivity factor, UL44, E3 SUMO ligase, PIAS3, SUMOylation, SUMO conjugation motif (SCM), subnuclear localization, ND10

## Abstract

Lytic replication of human cytomegalovirus (HCMV), a member of β-herpesvirus, is a highly complicated and organized process that requires its DNA polymerase processivity factor, UL44, the first-reported HCMV replication protein subjected to SUMO post-translational modification (PTM). SUMOylation plays a pleiotropic role in protein functions of host cells and infecting viruses. Particularly, formation of herpesviral replication compartments (RCs) upon infection is induced in proximity to ND10 subnuclear domains, the host cell’s intrinsic antiviral immune devices and hot SUMOylation spots, relying just on SUMOylation of their protein components to become mature and functional in restriction of the viral replication. In this study, to unveil the exact role of SUMO PTM on UL44 involved in HCMV replication, we screened and identified PIAS3, an annotated E3 SUMO ligase, as a novel UL44-interacting protein engaged in cellular SUMOylation pathway. Co-existence of PIAS3 could enhance the UBC9-based SUMO modification of UL44 specifically at its conserved ^410^lysine residue lying within the single canonical ψKxE SUMO Conjugation Motif (SCM). Intriguingly, we found this SCM-specific SUMOylation contributes to UL44 co-localization and interaction with subnuclear ND10 domains during infection, which in turn exerts an inhibitory effect on HCMV replication and growth. Together, these results highlight the importance of SUMOylation in regulating viral protein subnuclear localization, representing a novel way of utilizing ND10-based restriction to achieve the self-controlled slower replication and reproduction of herpesviruses.

## Introduction

Human cytomegalovirus (HCMV), a member of β-herpesvirus, mainly causes in neonates the congenital mental retardation and sensorineural hearing loss, as well as in immunodeficient individuals (e.g., transplant recipients, AIDS patients) several life-threatening complications [1]. One of the most central events in HCMV life cycle is the replication of its double-stranded genomic DNA, which takes place in the infected nuclei. The virus starts its genome replication from a single cis-acting origin, namely “oriLyt” that is flanked by auxiliary sequences required for maximal replication [2]. Viral DNA synthesis is then accomplished by a set of essential and specific HCMV proteins, such as UL44, UL54, UL70 (primase), *etc* [3]. Reported as a homodimeric accessory processivity factor, UL44 binds both DNA and UL54, the viral DNA polymerase, to stimulate the continuous viral genome synthesis [4]. The UL44 processivity activity toward UL54 resides in the N-terminus, since deletion of the C-terminal region (291–433 aa) does not affect its biochemical activities *in vitro* [5]. Crystal structure of the N-terminus (1–290 aa, two-thirds of the UL44 full length) is remarkably analogous to those of other DNA polymerase processivity factors like the sliding clamp PCNA in eukaryocyte and the protein UL42 in herpes simplex virus-1 (HSV-1) [6,7]. Moreover, UL44 was found to interact with multiple viral replication proteins through its N-terminal region (1–290 aa) [8]. In contrast, as to UL44 C-terminal part (291–433 aa), the exact biological role is still not fully understood, but this segment contains a nuclear localization signal (NLS) and phosphorylation of the three ^413, 415, 418^ Serine residues upstream of the NLS is indispensable for UL44 intranuclear localization and viral replication in HCMV-infected cells [9–11].

Post-translational modifications (PTMs), such as the above-mentioned phosphorylation but not limited to it, act as a universal means for cells and viruses to modulate their protein activities or interactions. In particular, SUMOylation, the covalent linkage of a small ubiquitin-related modifier (SUMO) to certain protein substrate, is an important kind of PTM essential for diverse cellular or viral protein functions, including the protein subcellular localization, protein–protein interaction, transcriptional regulation, DNA repair and maintenance of protein stability, etc [12,13]. At present, there are mainly three different isoforms of SUMO molecules described in mammals: SUMO-1 shows 47% amino acid identity to SUMO-2/-3 while SUMO-2 and SUMO-3 exhibit 95% homology to each other [14]. Similar to the ubiquitylation system, eukaryotic SUMOylation machinery also consists of three types of enzymes, except that in a SUMOylation reaction, the two ingredients of SUMO-activating enzyme E1 (uba2/aos1) and SUMO-conjugating enzyme E2 (UBC9) are strictly indispensable whereas the SUMO ligase E3 seems not always necessary [15]. Typically, modification of a SUMO molecule with its C-terminus onto the substrate protein selectively occurs at the lysine residue located within the ψKxE consensus motif (ψ is usually a hydrophobic residue; x is any residue), namely short SUMO conjugation motif (SCM) [16], but SUMOylations at non-SCM sites have also been observed. As for herpesviruses, the proteins undergoing SUMOylated regulation during infection generally belong to immediate-early ones, such as HCMV IE1 [17,18], HCMV IE2 [19,20], HSV-1 ICP0 [21] and human herpes virus-6 IE1 [22]. Nevertheless, UL44 was first characterized to be a HCMV DNA replication protein subjected to SUMOylation yet not belonging to immediate-early ones [23].

During HCMV and other DNA virus infections, viral DNA replication compartments (RCs), where early viral transcription and replication can be detectable [24,25], are often formed in proximity to the nuclear subcompartments of host cells termed as promyelocytic leukemia nuclear bodies (PML-NBs), or named nuclear domain 10 (ND10) compartments [26,27]. Appearing as dot-like discrete foci within the nucleoplasm, these PML-NB/ND10 subnuclear structures represent the multiprotein complexes of tumor suppressor PML, chromatin remodeling factor hDaxx, transcriptional regulator Sp100 and so on [26]. Based on bearing host restriction factors (e.g., PML, Sp100B/Sp100C/HMG/p53 *etc*.), one crucial ND10 function has been well known as part of the host cell intrinsic immunity against viral infection, despite the ultimate counteraction by viral early regulatory proteins like ICP0 in HSV-1 or IE1/pp71 in HCMV [28,29]. To this end, it’s challenging for DNA viruses during infection to establish their genome RCs close to such cellular antiviral ND10 structures, leaving the prerequisite to this RC-ND10 proximity an enigma.

In a recent HAdV (human adenovirus) study, one such prerequisite was pointed to site-specific SUMOylation at typical SCMs on virus, which was revealed to mediate an interaction of viral DNA-binding protein (E2A) in RC with the host ND10 scaffold protein PML [30]. In particular, ND10 assembly and integrity are dependent on SUMOylation of the scaffold protein PML and the resulting recruitment of other PML-interacting but still SUMOylated components (like hDaxx, Sp100) [31–33]. Moreover, another key functionality of ND10 domains in cell nuclei serves as hot SUMOylated reaction spots carrying a variety of SUMO-modifying enzymes and SUMO peptides [34], and even the large majority of ND10-associated proteins are also SUMO-linked [33]. Thus, given these enlightenments, we were prompted to question whether the SUMOylation of HCMV UL44 protein might also contribute to its association with the cellular ND10 structures during infection, and how this virus-host interplay would influence the viral DNA replicative capability. In fact, Sinigalia *et al*. first reported the UBC9-dependent SUMO modifications of UL44 occurring at multiple target lysine sites throughout the protein, yet without elucidation of the SUMOylation effects on UL44 due to protein expression issue after mutagenesis [23]; this is distinct from the above SCM site-specific SUMOylation of HAdV E2A protein that mediates the RC-ND10 proximity and interaction.

Until recently, we found ^410^lysine within a canonical SCM (ψ^410^KxE) site, located adjacent to the NLS motif of UL44, is the protein’s major SUMO modification site, which has no impacts on UL44 subcellular localization or protein stability but can attenuate the viral DNA replication upon its SUMOylation [35]. However, the selective mechanism by which SUMO is preferentially modified at K410 residue of UL44, as well as how this SCM-specific SUMOylation on UL44 can negatively regulate HCMV replication and reproduction, still remains unclear. In this study, to find more clues to regulatory role of SUMOylation on UL44, we performed a YTH (yeast two-hybrid) screening to identify potential UL44-interacting proteins, focusing on the members of cellular SUMOylation system. The results showed that besides the SUMO-conjugating enzyme UBC9, an annotated E3 SUMO ligase, PIAS3 (Protein Inhibitor of Activated STAT3), was also screened as a novel UL44 binding partner, and the UL44–PIAS3 interaction was further confirmed by a combination of co-immunoprecipitation, GST/His pull-down, HCMV-infection and fluorescent co-localization assays. Intriguingly, we next observed PIAS3 could enhance the SUMOylation of UL44, just specifically at that conserved ^410^lysine within the protein’s single canonical SCM site. This site-specific SUMOylation at SCM was revealed to guide the viral polymerase processivity factor UL44 to co-localize and interact with cellular ND10 structures, which in turn exerted a restriction on HCMV replication. Our work highlights that SUMOylation plays a critical regulatory role in subnuclear localization of the viral genomic replication protein, serving as a novel way of utilizing ND10-based restriction to achieve herpesviral self-controlled slower reproduction.

## Materials and methods

### Cells and viruses

293 T cells (Human Embryonic Kidney 293 T cells), U251 MG cells (Human astrocytoma U251 cells) and HFF cells (Human foreskin fibroblasts, passages 9 to 14) were cultured in DMEM (Dulbecco’s modified Eagle medium) containing 10% FBS (Fetal bovine serum). U251-PIAS3 cells (capable of stably overexpressing PIAS3) and U251-C cells (Control cell lines) were generated by transfecting U251 MG cells with the pcDNA3.1-PIAS3 construct and with the empty pcDNA3.1(+) vector (Invitrogen, Carlsbad, CA, USA), respectively. Then the positive single clone cells were identified by Western blot, following random resistance screening by G418 (Sigma-Aldrich, 600 μg/mL) in DMEM, and the constructed cell lines was continually maintained in medium supplemented with 200 μg/mL G418.

wt-HCMV (wild-type Towne strain, generated from Towne-_GFP-BAC_) was an appreciated gift from Dr. Fenyong Liu (University of California, Berkeley, CA, USA) [36]. HCMV derivative strains, v-K410R (introducing wt-HCMV with K410R mutation of UL44) and v-K410rev (the revertant wild-type virus for v-K410R), were constructed by a BAC (bacterial artificial chromosome)-based recombineering method, as described previously [37]. Briefly, in the first round of homologous recombination, a kana/str cassette, in possession of both the kanamycin and streptomycin resistance genes, was amplified by PCR to replace the UL44 ORF (open reading frame). The 69-nt forward or reverse primer is constituted by 50-nt UL44 homologous sequence in fusion with 19-nt kana/str cassette homologous sequence, both constituent fragments simultaneously from the 5ʹ or 3ʹ ends of their target sequences: 5ʹ-ATGGATCGCAAGACGCGCCTCTCGGAGCCACCGACGCTGGCGCTGCGGCTTCGCTGTCGAGATATGACG-3ʹ; 5ʹ-CTAGCCGCACTTTTGCTTCTTGGTGTTAGGGACGAACTCGAACGTTACAGGTATTGGCCCCAATGGGGT-3ʹ. Subsequently, the Towne BAC-bearing EL350 strain was transformed with the above PCR product and the recombinant was selected via kanamycin resistance. In the second round of recombination, wild type or K410R mutant of *UL44* gene was amplified by PCR from pCMV-Myc-UL44 or pCMV-Myc-UL44-K410R, using the primers: 5ʹ-ATGGATCGCAAGACGCGCCTCTCGGAGCCACCGACGCTGGCGCTGCGGCT-3ʹ; 5ʹ-CTAGCCGCACTTTTGCTTCTTGGTGTTAGGGACGAACTCGAACGTTACAG-3ʹ. The bacteria carrying the rescued BACs were screened through the resistance of streptomycin. wt-HCMV, v-K410R and v-K410rev were proliferated in HFF cells, and the viral stocks were preserved at −80°C in DMEM containing 10% FBS and 1.5% BSA (bovine serum albumin).

### Reagents and antibodies

G418 reagent was obtained from Sigma-Aldrich (St. Louis, MI, USA). The purchased commercial antibodies in this study are listed as follows: Mouse monoclonal anti-Myc 9E10 antibody (M4439, Sigma), Mouse monoclonal anti-Flag (M2) antibody (F3165, Sigma), Rabbit polyclonal anti-Flag antibody (F7425, Sigma), Rabbit polyclonal anti-HA antibody (H6908, Sigma), Rabbit polyclonal Anti-human PIAS3 antibody (ab58406, Abcam, Cambridge, UK), Rabbit monoclonal Anti-PML Protein antibody (ab179466, Abcam), Rabbit monoclonal anti-UBC9 antibody (ab75854, Abcam), Mouse mAb against HCMV UL44 (sc-69,744, Santa Cruz Biotechnology, Heidelberg), Mouse anti-HCMV UL84 mAb (sc-56,977, Santa Cruz); Mouse anti-His mAb (66,005-1-Ig, Proteintech, Rosemont, USA), Mouse anti-GST mAb (66,001-2-Ig, Proteintech), Mouse anti-GAPDH mAb (60,004-1-Ig, Proteintech); Dylight 488/549/649-conjugated secondary antibodies, used for immunofluorescence, were from Abbkine, USA. In addition, His-tagged UL70 protein was expressed and purified as described before, and the corresponding rabbit polyclonal antibody against HCMV UL70 was then prepared in our laboratory [38,39].

### Plasmid construction

The plasmids constructed in this study are listed in Table 1. Full-length ORF of HCMV UL44 was first amplified by PCR from the BAC of HCMV Towne strain, and then cloned into pCMV-Myc (Clontech) digested by SalI/KpnI to yield the plasmid pCMV-Myc-UL44 as well as into pET-28a (Novagen) digested by NdeI/BamHI to generate the pET-28a-UL44. Similarly, full-length PIAS3 coding sequence was amplified by PCR from the human cDNA library. The plasmids pGEX-PIAS3 (using pGEX-6p-1vector), pRK-Flag-PIAS3 (using pRK11-Flag vector) and pcDNA-His-PIAS3 (using pcDNA3.1(+) vector) were then individually constructed for the protein expression of prokaryotic GST-tagged PIAS3, mammalian Flag-tagged PIAS3 and mammalian His-tagged PIAS3, respectively. Additionally, QuikChange mutagenesis kit (Stratagene) was used to generate the corresponding derivative plasmids below: pCMV-Myc-UL44-K410R (replacing ^410^lysine of UL44 with ^410^arginine), pCMV-Myc-UL44wt-ΔNLS (expressing UL44 mutant without NLS motif at 425–432 aa), pcDNA-HA-UBC9-C93S (mutating ^93^cysteine of UBC9 to ^93^serine) and pcDNA-His-PIAS3-C334S (encoding the C334S variant of PIAS3). Besides, pRK-Flag-SUMO-1/2/3 was also constructed to express the active Flag-tagged SUMO-1, −2 or −3 peptide. The accuracy of each plasmid generated here was confirmed by the profile of restriction digestion and DNA sequencing.

**Table 1.** The plasmids constructed in this study

Plasmids	Description	Reference /Source
pCMV-Myc-UL44	pCMV-Myc containing HCMV UL44 full-length coding region	This study
pCMV-Myc-UL44-K410R	pCMV-Myc containing HCMV UL44 sequence with the K410R mutation	This study
pCMV-Myc-UL54	pCMV-Myc containing HCMV UL54 full-length coding region	This study
pCMV-Myc-UL44wt-ΔNLS	pCMV-Myc containing HCMV UL44 sequence without NLS motif (425–432 aa)	This study
pET28a-UL44	pET28a containing HCMV UL44 full-length coding region	This study
pRK-Flag-PIAS3	pRK11-Flag containing PIAS3 cDNA full-length sequence	This study
pcDNA-His-PIAS3	pcDNA3.1(+)-His containing PIAS3 full-length sequence	This study
pcDNA-His-PIAS3-C334S	pcDNA3.1(+)-His containing PIAS3 sequence with the C334S mutation	This study
pRK-Flag-SUMO-1/2/3	pRK11-Flag containing SUMO-1, −2 and −3 sequence respectively.	This study
pGEX-PIAS3	pGEX-6p-1 containing PIAS3 full-length sequence	This study
pGEX-PIAS3-C334S	pGEX-6p-1 containing PIAS3 sequence with the C334S mutation	This study
pcDNA-HA-UBC9	pcDNA3.1(+)-HA containing UBC9 full-length sequence	This study
pcDNA-HA-UBC9-C93S	pcDNA3.1(+)-HA containing UBC9 sequence with the C93S mutation	This study

### Yeast Two-Hybrid Screening

Human fetal brain cDNA library, constructed on the pACT2 vector for fusion expression of the GAL4 activation domain, and *Saccharomyces cerevisiae* AH109 strain were purchased from Clontech (Mountain View, CA, USA). Firstly, to exclude the self-activation of report genes by UL44 protein alone, AH109 strain was co-transformed with pGADT7 empty vector carrying the GAL4 activation domain as well as pGBKT7-UL44 plasmid harboring the DNA-binding domain of GAL4, followed by growth screening on QDO (synthetic dropout of four nutrients, namely deficient in adenine, histidine, leucine and tryptophan) medium and tests of β-galactosidase activity. Next, AH109 yeast cells were co-transformed with both pGBKT7-UL44 and the cDNA library plasmids. Positive clones were similarly selected on QDO medium and assayed on β-galactosidase activity. From the positive yeast colonies, *E.Z.N.A*. Yeast Plasmid Kit (Omega Bio-Tek) was used to extract the corresponding cDNA library plasmids that were later rescued by introduction into *E. coli DH5a*. Meanwhile, co-transformation of AH109 strain with pGBKT7 empty bait vector and the cDNA library plasmids was also set up as a negative control. Lastly, the inserted cDNAs on library plasmids were identified by DNA sequencing using the GAL4AD primer (5ʹ-AATACCACTACAATGGAT-3ʹ) and Homology retrieval on NCBI (National Center for Biotechnology Information) database.

### Co-immunoprecipitation

To check the UL44–PIAS3 interaction, both pCMV-Myc-UL44 and pRK-Flag-PIAS3 plasmids were co-introduced into 293 T cells. After 48 hours, NP40 lysis buffer containing protease inhibitor cocktail (Roche) was added to collect the supernatant of cell lysate by centrifugation. ProFound Mammalian IP/Co-IP kit (Pierce) was then utilized to perform Co-IP (co-immunoprecipitation) experiments as the manufacturer instructed. The efficient antibody crosslinking onto Protein A/G magnetic beads was accomplished by Pierce Crosslink Magnetic IP/Co-IP Kit. The UL44/PIAS3 proteins immunoprecipitated by anti-Myc or anti-Flag antibody were detected through Western blot. Besides, Co-IP assays of UL44 with PIAS3 or with PML in HCMV-infected cells were carried out likewise as above.

### Protein purification

Protein expression and purification were conducted with reference to the previous method [40]. *E. coli* BL21 (DE3) transformants of pET28a-UL44 or pGEX-PIAS3 were individually cultured at 37°C in LB medium (containing 50 μg/mL kanamycin or 100 μg/mL ampicillin) until the OD_600_ reached 0.6, before subsequent induction of protein expression by 0.2 mM IPTG (isopropyl-β-D-thiogalactopyranoside) at 16°C for 22 h. Harvested by centrifugation (4,000 rpm for 20 min at 4°C), the induced bacteria were resuspended in pre-cooled Buffer I (25 mM Tris-HCl pH 7.4, 300 mM NaCl), and subjected to cell lysis by sonication on the ice (working at 100 W for 5 s and intermission for 10 s, 60 cycles). Next, the lysis supernatant was collected by centrifugation (12,000 rpm for 70 min at 4°C) and applied to a Ni^2+^-NTA or GST-binding resin column (Qiagen) pre-balanced with Buffer I. After a 20-min incubation at 4°C, unbound proteins flowed through the column, and nonspecifically bound proteins were washed off the column using sufficient Buffer I (supplemented with 20 mM imidazole for the washing of nickel column). Then the resin-specific binding target proteins, His-tagged or GST-tagged, were eluted by Buffer II (25 mM Tris-HCl pH 7.4, 150 mM NaCl and 300 mM imidazole) or Buffer III (25 mM Tris-HCl pH 8.0, 150 mM NaCl and 20 mM reduced glutathione), respectively. Determined on 12% SDS-PAGE gels stained with CBB (Coomassie brilliant blue)-R250, the eluted fractions of target protein were mixed together and concentrated to 1.5 mL with a Millipore Centrifugal Ultra-Filter (10,000 MWCO, 15 mL), followed by further SEC (size exclusion chromatography) purification with a Superdex 200 increase column (GE Healthcare) using the Buffer SD (25 mM Tris-HCl pH 7.4, 150 mM NaCl and 1 mM DTT). At last, purified target proteins were dissolved in Buffer SD supplemented with 10% glycerol before storage at −80°C as 100 μL per aliquot for *in vitro* assays. Using BSA (bovine serum albumin) as a standard, the Bradford method was adopted to determine protein concentrations.

### GST/His pull-down assay

*E. coli* BL21 (DE3) transformants at exponential growth, harboring pGEX-PIAS3 or pET28a-UL44, were induced by IPTG for overexpression of N-terminal GST-tagged PIAS3 or N-terminal His-tagged UL44 fusion protein, which was later purified by nickel column or GST column affinity chromatography. Then, anti-GST or anti-His antibody-conjugated agarose beads were incubated with the protein mixture of purified GST-PIAS3 and His-UL44, before the washing with PBS-T. The proteins attached to the agarose beads were detected by Western blot with anti-His and anti-GST antibodies, respectively.

### Fluorescent localization

Cells to be observed, after three times of washing with PBS (adding 0.1% BSA), were *in situ* at 25°C subjected to fixation in 4% (w/v) paraformaldehyde for 20 min and to subsequent permeabilization with 0.2% TritonX-100 dissolved in PBS for 10 min. Then, following blocking with 5% BSA at 37°C for 1 h and the thorough washing with PBS, cells in confocal dishes were incubated with specific primary antibodies of certain species (1:100) and later with species-specific fluorescent secondary antibodies (1:1000), both conducted at RT (room temperature) for 1 h. Lastly, DAPI (Invitrogen) and 50% glycerol were used for nuclear staining and sealing, respectively. Confocal images were collected in separate channels from an Olympus FV1000 microscope installed with the Olympus Fluoview analysis software. The displayed images were representative ones from three independent experiments.

### *SUMOylation analysis* in vivo *or* in vitro

293 T cells were co-transfected with pCMV-Myc-UL44 or pCMV-Myc-UL44-K410R, pcDNA-His-PIAS3 (wild-type or C334S mutant) and other SUMOylation-related plasmids (pRK-Flag-SUMO-1/-2/-3, pcDNA-HA-UBC9) as indicated using Lipofectamine 2000 (Invitrogen). After 48 h, the *in vivo* SUMO modifications of UL44 and its K410R mutant in transfected cells were analyzed by Western blot using anti-Myc and anti-Flag antibodies, as described in our previous work [35]. Meanwhile, to analyze the SUMOylation of UL44 under HCMV infection, U251 cells were first transfected with siControl or siPIAS3, and 24 h later infected with wt-HCMV or v-K410R at an MOI of 1. At 48 hpi (hour post infection), total cell lysates were immunoprecipitated with anti-UL44 antibody or analyzed directly by SDS-PAGE, followed by detection of UL44 and UL44-SUMO proteins using anti-UL44 or anti-SUMO-1 antibody. Additionally, *in vitro* SUMOylation assays were performed with the SUMOylation Kit from Enzo Life Science (Catalog #BML-UW8955) according to the manufacturer’s suggestions.

### Transient transfection of siRNA into cells

siPIAS3 (5ʹ-GGUCGAAGUUAUUGACUUG-3ʹ) [41], a chemically synthesized siRNA that specially targets the CDS region of PIAS3 mRNA, as well as siControl (a negative control siRNA molecule), was supplied from Ribobio Co. Ltd (Guangzhou, China). Then, following the manufacturer’s instructions, U251 cells were transfected with the siRNAs (siPIAS3 or siControl) by Lipofectamine^TM^ 2000 (Invitrogen).

### Construction of RNAi-transduced stable cells

The construction was performed as previously described [37]. Briefly, Lentiviruses were first produced in 293 T cells by co-transfection of two packaging plasmids (pMD2G and psPAX2) plus a control or a RNAi pLKO.1 lentiviral plasmid, using the Lipofectamine^TM^ 2000 (Invitrogen). Then, well-packaged viral particles were collected and concentrated for infection of the target U251 cells in 6 cm plates, and selection of the stably transduced cells was achieved by supplementing the culture medium with 2 μg/mL Puromycin. Here, the sequence for UL54 shRNA was 5ʹ-CTGCTCAACAAGTGGGTTT-3ʹ (siUL54) [42], and the targeting sequence for PML shRNA was 5ʹ-AGATGCAGCTGTATCCAAG-3ʹ (siPML) [43]. Silencing of the corresponding gene expression was confirmed by Western blot.

### Real-Time qPCR of HCMV DNA levels

The DNeasy tissue kit (Qiagen, Hilden, Germany) was adopted to extract total DNA from mock or HCMV-infected cells. Quantification of intracellular viral DNA levels was accomplished by Real-Time PCR amplification of the HCMV UL83 DNA region, using a pair of primers plus a specific TaqMan probe [37]. The copies of viral genome were normalized to those of the cellular RNase P using the primers and probe reported before [37]. Three times of repetitions were carried out for each set of assays in triplicate. The shown data are Means ± SD from one representative experiment.

### Determination of viral growth curves

To determine the curves of viral growth, U251 cells of 95% confluency were either mock- or HCMV-infected at an MOI of 0.5 using the DMEM containing 1% FBS. After 2-h incubation for virus absorption, the medium was replaced with DMEM containing 10% FBS, in which cells were continually cultured at 37°C until collected at the indicated infection time points by scraping. Then, to obtain the total proliferating viruses, the infected cells as well as medium were mixed with an equal volume of 10% (w/v) skimmed milk, followed by three repeated freeze-thaw cycles and removal of cell debris by centrifugation (3000 g for 30 min at 4°C) to collect the supernatant as viral stocks. Finally, standard virus plaque assays were performed in HFF cells to measure the titers of viral stocks based on the counted numbers of viral plaques after 14-day infection [44]. The titer values denoted the means ± SD from three repeated experiments.

### Statistical analysis

The GraphPad Prism 6.0 software was applied to all statistical analyses. At least three repetitions were conducted for each experiment, in which each sample was set in triplicate. Data represented the Average ± SD (standard deviation) from three assays. The Averages, between 2 groups or among more (≥3) groups, were appraised by usage of unpaired Student’s *t*-test or one-way ANOVA (analysis of variance) with Tukey’s *post hoc* test. NS denoted no significant difference. Differences were considered statistically significant when * denoted p < 0.05, ** denoted p < 0.01, and *** denoted p < 0.001.

## Results

### Identification of PIAS3 as a UL44-interacting protein

To identify cellular proteins interacting with HCMV UL44, we adopted a GAL4 yeast Two-Hybrid System (CloneTech) to screen up to 2 × 10^7^ independent clones from a human fetal brain cDNA library with the full-length UL44 protein as bait. 56 individual positive yeast colonies were isolated, displaying strong and specific interaction with UL44. Subsequent sequence analyses showed that 10 out of the 56 clones encode either the full-length or partial region of the human SUMO E3 ligase PIAS3 (Table 2).

**Table 2.** Interactions detected in the Y2H screening of HCMV UL44 against the human fetal brain cDNA library

CMV Bait	Prey Gene Symbol	Prey Gene ID	Prey Gene Name	Number of hits	Primary Function	Secondary Function	Notes
**UL44**	EXOSC9	5393	exosome component 9	2	exoribonuclease activity	RNA processing	
	PFAAP5	10,443	phosphonoformate immuno-associated protein 5	1	PFA-stimulus protein?		confirmed by coIP
	**PIAS3**	**10,401**	**protein inhibitor of activated STAT, 3**	**10**	**SUMO ligase activity**	**transcriptional regulator**	**confirmed by coIP**
	RANBP9	10,048	RAN binding protein 9	1	translocation of RNA and protein	signal transduction	not confirmed by coIP
	SRI	6717	sorcin	1	calcium channel regulator	signal transduction	
	SAMM50	25,813	sorting and assembly machinery component 50 homolog (S. cerevisiae)	3	mitochondrial membrane assembly		
	TDG	6996	G/T mismatch-specific thymine DNA glycosylase	2	DNA base-excision repair		not confirmed by coIP
	**UBE2I**	**7329**	**ubiquitin-conjugating enzyme E2I (UBC9 homolog, yeast)**	**36**	**ubiquitin cycle/SUMOylation cycle**	**protein modification**	**confirmed by coIP**

PIAS3 is a member of the mammalian PIAS family, which also includes PIAS1, PIASxα/β, and PIASy [45]. This family of proteins, sharing over 40% sequence homology, possess four conserved structural elements: PINIT motif, SIM motif (SUMO-interacting motif) for SUMO binding, SAP domain at N-terminus and SP-RING domain for E3-SUMO ligation. PIAS3 was originally characterized to be an inhibitor of activated STAT3 pathway [46,47] and later to be a co-regulatory protein for other transcriptional regulators such as Gfi-1 [48], metastasis tumor antigen 1 (MTA1) [49], DBC1 [50], *etc*. in various cellular pathways, both functions of which rely most likely on its SUMO-conjugating activity.

**Figure 1.**
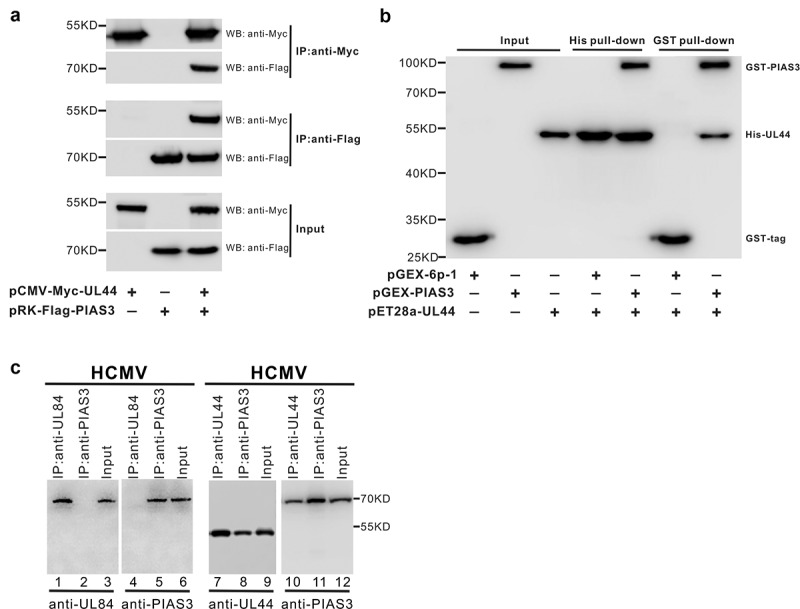
**UL44, HCMV DNA polymerase processivity factor, interacts with human E3 SUMO ligase PIAS3**. **(a)** Co-IP analysis of the association between UL44 and PIAS3 in transfected cells. pCMV-Myc-UL44 and pRK-Flag-PIAS3 plasmids were co-introduced into 293 T cells. At 48 h post transfection, the supernatant of cell lysate was taken to IP (immuno-precipitate) with anti-Myc or anti-Flag mAbs. The immunoprecipitants, after heat denaturation, were analyzed by Western blot with anti-Myc or anti-Flag mAbs. Meanwhile, negative controls were set up by cell co-transfection with pCMV-Myc-UL44 and a plasmid expressing the Flag tag, or with pRK-Flag-PIAS3 and a plasmid expressing the Myc tag. **(b)** His and GST pull-down analyses of the direct interaction between UL44 and PIAS3. Expressed in *E. coli* BL21 (DE3), His-tagged UL44 and GST-tagged PIAS3 were individually purified by affinity chromatography using Ni^2+^-NTA or GST column. Then, the reciprocal GST and His pull-down assays were performed to verify the direct UL44-PIAS3 interaction *in vitro*. **(c)** Co-IP analysis of UL44 association with endogenous PIAS3 under HCMV infection. U251 cells were infected with wt-HCMV at an MOI of 1, before preparation of the total cell lysates at 48 hpi. The input proteins and IP samples were analyzed by Western blot, using as the indicated anti-UL84, anti-UL44 or anti-PIAS3 antibody, respectively. For all panels, results were representative of three independent experiments

To validate the interaction between UL44 and PIAS3 screened from the YTH, co-immunoprecipitation assays were first performed to determine whether it could also occur in human cells. cMyc-UL44 and Flag-PIAS3 were transiently co-expressed in HEK293T cells, and the cell lysates were incubated with immobilized anti-Myc or anti-Flag agarose beads to isolate the corresponding Myc/Flag-tag protein complexes. Subsequent Western blot analyses with Myc/Flag antibodies suggested that Myc-UL44 and Flag-PIAS3 could co-immunoprecipitate with each other in a specific manner (Figure 1a). Next, the direct interaction of UL44 with PIAS3 was continually verified by *in vitro* pull-down assays. Prokaryotic expressed fusion proteins, His-UL44 and GST-PIAS3, were mixed with Ni-NTA or Glutathione Sepharose 4B affinity resins to enrich His-tagged or GST-tagged protein complexes detected by Western blot. As shown in Figure 1b, His-UL44 specifically pulled down the GST-PIAS3, and vice versa. Furthermore, the physiological association of UL44 with PIAS3 during HCMV infection was also directly demonstrated in HCMV-infected U251 cells with native antibodies (non-tagged) (Figure 1c). Therefore, PIAS3 was screened out to be a novel SUMO-related protein capable of direct interaction with UL44 during HCMV infection of human cells.

### Nuclear co-localization of UL44 and PIAS3 in human cells

**Figure 2.**
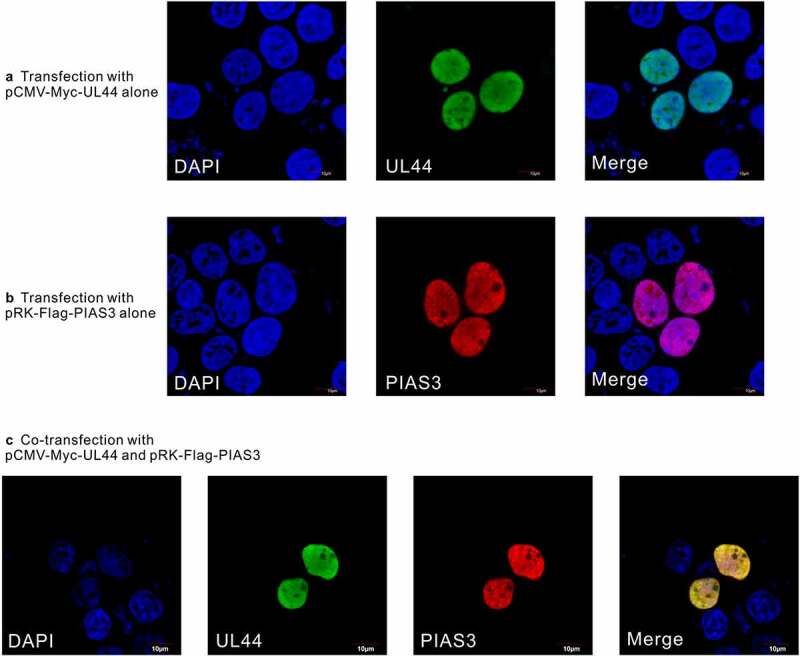
**Co-localization of UL44 with PIAS3 in human transfected cells**. U251 cells were transfected individually with **(a)** the single pCMV-Myc-UL44 construct, **(b)** the single pRK11-Flag-PIAS3 construct, or **(c)** both of pCMV-Myc-UL44 and pRK11-Flag-PIAS3 together. At 48 h post transfection, cells were fixed and permeabilized, followed by incubation with anti-Flag/anti-Myc primary Abs and with Dylight 488/549-conjugated fluorescent secondary Abs. The subcellular localization of UL44 (green) and PIAS3 (red) were visualized, with the cell nuclei stained by DAPI (blue). For a same field of view, “Merge” denoted the overlapping fluorescence from both the nuclei and expressed proteins. The displayed images were representative ones from three independent experiments

In order to check whether the observed interaction between UL44 and PIAS3 might influence the intracellular localizations of each other, we carried out confocal assays on UL44 and PIAS3 proteins in U251 cells. cMyc-UL44 and Flag-PIAS3 proteins were individually or together expressed in human U251 cells, respectively, followed by indirect immunofluorescence microscopy with mouse anti-Myc and rabbit anti-FLAG antibodies. As shown in Figure 2(a,b), both UL44 and PIAS3 were found to be mainly located in the cell nuclei when either of them was expressed alone with the corresponding construct of pCMV-Myc-UL44 or pRK11-FLAG-PIAS3, respectively. In comparison, co-transfection experiments showed that Myc-tagged UL44 and FLAG-tagged PIAS3 still co-localized predominately in the nucleus of U251 cell, without significant localization changes of both proteins (Figure 2c). This observation of UL44 co-localization to PIAS3 in human cell nuclei further supported the potential physiological interaction between UL44 and PIAS3 during HCMV infection.

### PIAS3 can effectively enhance the SUMOylation of UL44

The PIAS family proteins typically contain a central conserved RING-like finger domain, which is responsible for UBC9 (E2) binding and SUMO ligase activity (E3) [51]. Reminiscently, UL44 could be SUMOylated to a baseline level at multiple sites upon its interaction with UBC9 (E2) [23]. We thus wondered if PIAS3 could act as an additional E3 SUMO ligase to influence the SUMOylation of UL44. Inactivated PIAS3-C334S was accordingly set up as a control, in which the conserved ^334^cysteine residue in the RING-like finger domain was mutated to serine [52].

**Figure 3.**
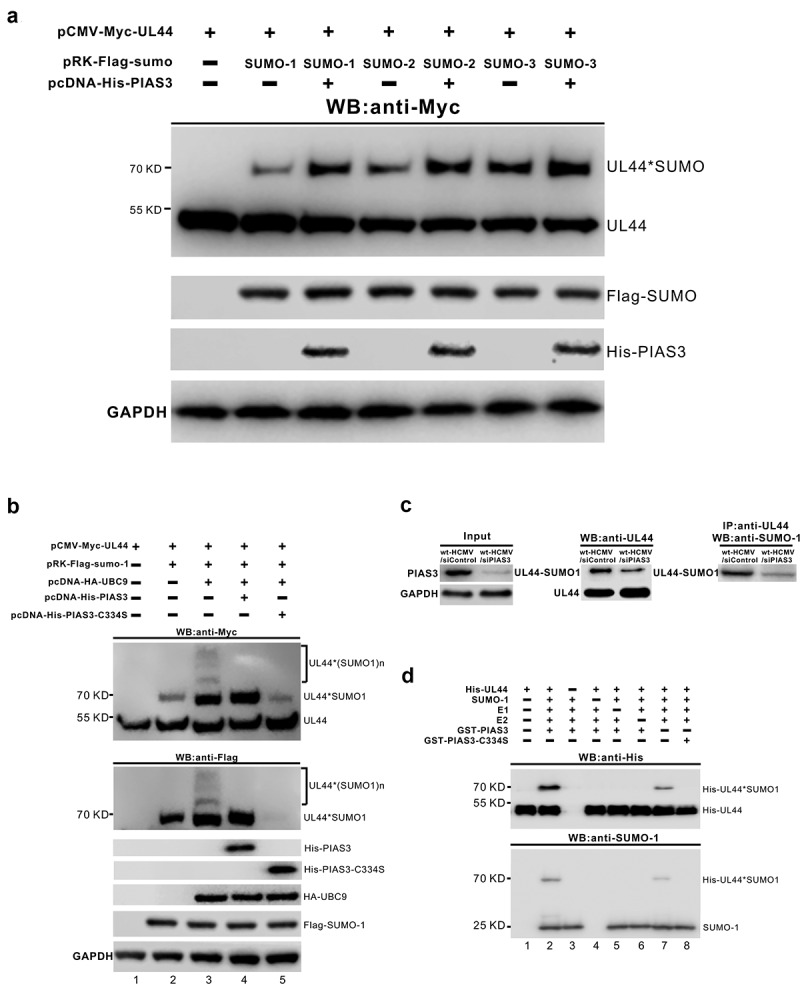
**PIAS3 exerts an enhancing effect on UL44 SUMOylation**. **(a)** The *in vivo* SUMOylation of UL44 in transfection assays, in the presence of exogenous PIAS3 using different SUMO peptides. pCMV-Myc-UL44 and pRK-Flag-SUMO-1/2/3, along with or without pcDNA-His-PIAS3, were co-introduced into HEK293T cells. At 48 h post transfection, Western blot with anti-Myc antibody was performed to detect UL44 and UL44-SUMO in total cell lysates. Free SUMO-1/2/3 peptides were probed with anti-Flag antibody. GAPDH was set as the internal control. **(b)** The *in vivo* SUMOylation of UL44 in transfection assays, in the presence of exogenous UBC9 and PIAS3 (WT or C334S mutant). pCMV-Myc-UL44, pRK-Flag-SUMO-1 and pcDNA-HA-UBC9, along with or without pcDNA-His-PIAS3 (WT or C334S mutant) were co-introduced into HEK293T cells. At 48 h post-transfection, Western blot with anti-Myc or anti-Flag antibody was performed to analyze UL44 and UL44-SUMO in total cell lysates. SUMO-1, UBC9 and PIAS3/PIAS3-C334S were probed with anti-Flag, anti-HA and anti-His antibodies, respectively. GAPDH served as the internal control. **(c)** The *in vivo* SUMOylation of UL44 during wt-HCMV infection, upon interference of endogenous PIAS3. U251 cells, after transfection with siControl (a negative control siRNA molecule) or siPIAS3 (a chemically synthesized siRNA that specially targets the CDS region of PIAS3 mRNA) for 24 h, were infected with wt-HCMV at an MOI of 1. At 48 hpi, the supernatant of total cell lysates, either immunoprecipitated with anti-UL44 antibody or not, was analyzed by Western blot using anti-UL44 or anti-SUMO1 antibody as indicated to detect UL44 and UL44-SUMO proteins. **(d)** The *in vitro* SUMOylation assays on UL44 protein. His-UL44, GST-PIAS3 and GST-PIAS3-C334S were individually expressed in *E. coli* BL21 (DE3), before further protein purification by affinity chromatography and SEC (size exclusion chromatography). Enzymatic reactions were set up with the SUMOylation-related components as indicated, and the production of SUMOylated UL44 was determined by Western blot with anti-His or anti-SUMO1 antibody. For all panels, each set of assays was repeated three times and a representative one was shown

We first examined the E3 SUMO ligase activity of PIAS3 toward UL44 *in vivo*, using different SUMO substrates or siRNA regulators. HEK293T cells were co-transfected with pCMV-Myc-UL44 and one of the serial SUMO expression vectors (pRK-Flag-SUMO-1, 2 or 3), in the absence or presence of wild-type/inactive PIAS3. Surprisingly, Western blots of total cell lysates after a 48-h culture showed that presence of PIAS3 could render all the SUMO substrates (SUMO-1, 2 and 3) more efficiently conjugated to UL44, without distinct discriminations among the three SUMO proteins (Figure 3a). In contrast, the inactive PIAS3-C334S mutant, when co-expressed with SUMO-1 substrate in 293 T cells, lost the ability to enhance SUMO-1 conjugation to UL44 (Figure 3b, lane 5), relative to the case in wild-type PIAS3 (Figure 3b, lane 4). Moreover, upon down-regulation of the endogenous PIAS3 via a specific short hairpin RNAs (siPIAS3), the intracellular UL44-SUMO1 level in HCMV-infected cells became obviously decreased (Figure 3c). Of note, previous study showed that supraphysiological expression of UBC9 in transfected cells resulted in detection of several slower-migrating blot bands of UL44 bearing multiple SUMO conjugations at its different lysine receptor sites throughout the protein (Figure 3b, lane 3) [53]. Here, instead, co-presence of over-expressed PIAS3 generated mainly one band of SUMOylated UL44 with enhanced modifications (Figure 3b, lane 4), which implied the possibility of site-specific SUMO conjugation onto UL44 mediated by PIAS3.

Further, SUMOylation enzymatic assays were performed to directly verify the E3 SUMO ligase activity of PIAS3 toward UL44 *in vitro*. Purchased E1 SUMO-activating enzyme, UBC9 (E2) and SUMO-1, as well as purified His-UL44, were incubated in the absence or presence of GST-PIAS3/GST-PIAS3-C334S, followed by immunoblotting to detect the SUMOylation of UL44. As shown in Figure 3d, although the UL44-SUMO1 band could be observed with the minimal help of E1 and E2 (lane 7), its intensity was weaker than that produced via an additional incorporation of wild-type PIAS3 (lane 2). In particular, the C334S mutation of PIAS3 resulted in not only loss of its enhancement in UL44-SUMO1 level, but even inactivation of the baseline SUMOylation fulfilled by E1 and E2 (lane 8); this might be due to the potential interaction of PIAS3-C334S with E2 that disturbed the baseline SUMO modification reaction.

Briefly, our data revealed that based on the site-flexible baseline SUMOylation of UL44 catalyzed by E1 and UBC9 (E2), PIAS3 acts as an additional E3 SUMO ligase to effectively enhance the SUMOylation levels of UL44 using SUMO-1, 2 or 3 peptides.

### ^410^lysine within the single canonical SCM of UL44 serves as the specific SUMOylation-enhancing site regulated by PIAS3

**Figure 4.**
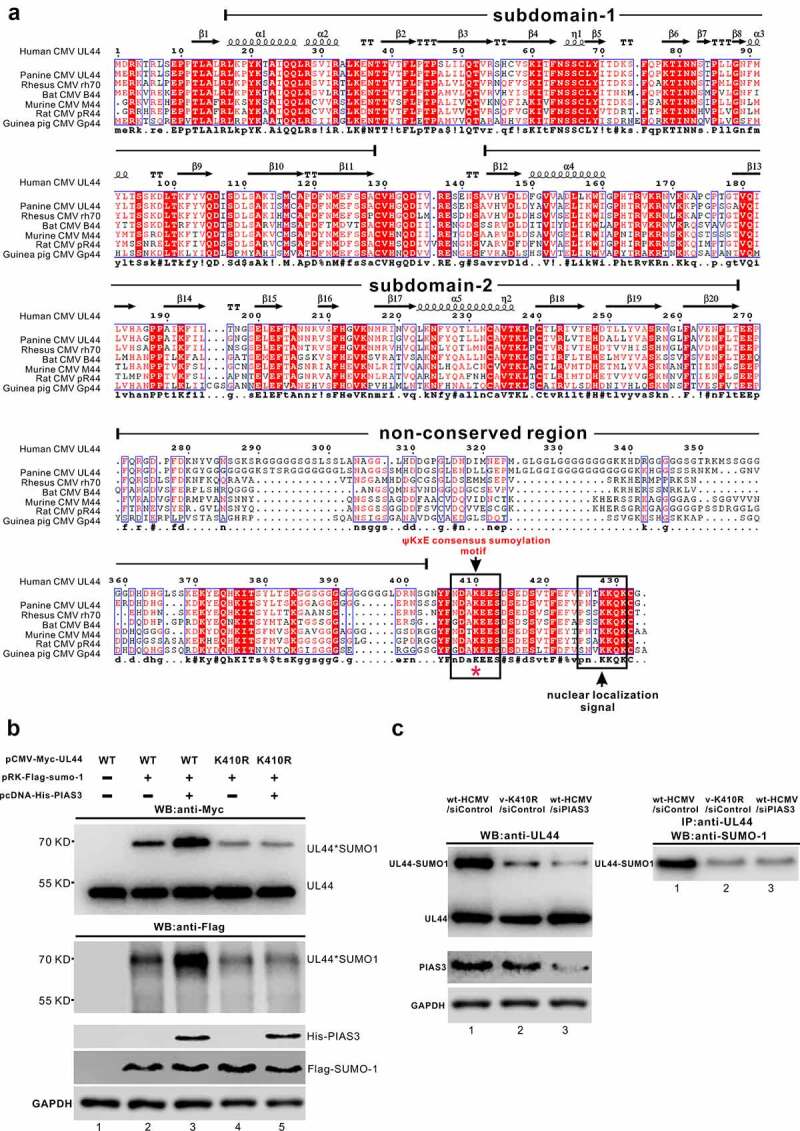
**The SUMOylation-promoting effect of PIAS3 on UL44 is site-specific at stringently conserved ^410^lysine residue within the single canonical SCM (SUMO Conjugation Motif; ψ^410^KxE)**. **(a)** The multiple sequence alignment among UL44 and other CMV homologs. Subdomain-1 and Subdomain-2 denote the two topologically conserved subdomains connected by a loop [7]. The two sequences indicated by black arrows and boxes, lying as a conserved region at the C-terminus, represent the single canonical SCM (SUMO Conjugation Motif; ψKxE) site and the NLS (nuclear localization signal), respectively. **(b)** Influence of the K410R mutation on SUMOylation of UL44 in transfection assays, in the presence of exogenous PIAS3. pCMV-Myc-UL44 (WT or K410R) and pRK-Flag-SUMO-1, along with or without pcDNA-His-PIAS3, were co-introduced into HEK293T cells. At 48 h post transfection, UL44 and UL44-SUMO proteins in total cell lysates were analyzed by Western blot with anti-Myc or anti-Flag antibody. PIAS3 and SUMO-1 were probed with anti-His and anti-Flag antibody, respectively. **(c)** The E3 SUMO ligase activity of endogenous PIAS3 toward UL44 during HCMV (WT or K410R mutant) infection. U251 cells, after transfection with siControl or siPIAS3 for 24 h, were infected with HCMV (WT or K410R mutant) at an MOI of 1. At 48 hpi, the supernatant of total cell lysates, either immunoprecipitated with anti-UL44 antibody or not, was analyzed by Western blot using antibodies as indicated (Left: anti-UL44; Right: anti-SUMO-1) to detect UL44 and UL44-SUMO proteins. The endogenous PIAS3 was probed with anti-PIAS3 antibody. In both (b) and (c), GAPDH served as the internal control and results were representative of three repeated experiments

We next sought to determine whether the SUMOylation-enhancing effect of PIAS3 on UL44 is site-specific as we hypothesized above, or it acts in a non-site-specific pattern like the SUMOylation fulfilled by E1 and UBC9 (E2). UL44 protein totally contains 31 lysines. Among them, using SUMOsp 2.0 prediction software [54], we previously found only one canonical SCM (SUMO Conjugation Motif; ψ^410^KxE) site at ^410^lysine, which functions as the major SUMOylation site of UL44 to attenuate HCMV replication [35]. Sequence alignments showed that this canonical ψ^410^KxE site is single and strictly conserved among UL44 and other CMV homologues, lying just adjacent to the UL44 NLS motif at C-terminal end (Figure 4a).

Considering the remarkable down-regulated effect of siPIAS3 on the UL44-SUMO level (Figure 3c), we were prompted to assess whether the major SUMOylation of UL44 at ^410^lysine within the single SCM site is directed by the SUMOylation-promoting role of PIAS3. Firstly, the influence of K410R mutation on UL44 SUMOylation was examined, in the absence or presence of exogenous PIAS3, in co-transfected HEK293T cells. As shown in Figure 4b, substitution of the major SUMOylated ^410^lysine residue with arginine led to a great decline in UL44-SUMO1 level (lane 4 versus lane 2). However, PIAS3 lost the capability to enhance UL44 SUMOylation at other lysine residues outside the SCM site, even unable to recover the SUMOylation level of K410R mutant to that of WT (lane 5 versus lane 4 in Figure 4b); this is distinct from UBC9, which could still partially recover the SUMOylation capability of UL44-K410A mutant [35]. In addition, HCMV infection assays also hinted the physiological correlation of UL44 SCM site with PIAS3. Once wt-HCMV (wild-type Towne strain virus) was mutated into v-K410R (BAC-derived HCMV with K410R substitution), E3 SUMO ligase activity of endogenous PIAS3 became inhibited toward the UL44-K410R mutant, while UL44-wt lost its majority of SUMOylated forms upon interference of endogenous PIAS3 (Figure 4c).

Together, the SUMOylation-enhancing role of PIAS3 is site-specific on UL44 at stringently conserved ^410^lysine within the single canonical SCM.

### Canonical SCM-specific SUMOylation of UL44 mediates its subnuclear co-localization and interaction with ND10 structures during HCMV infection

**Figure 5.**
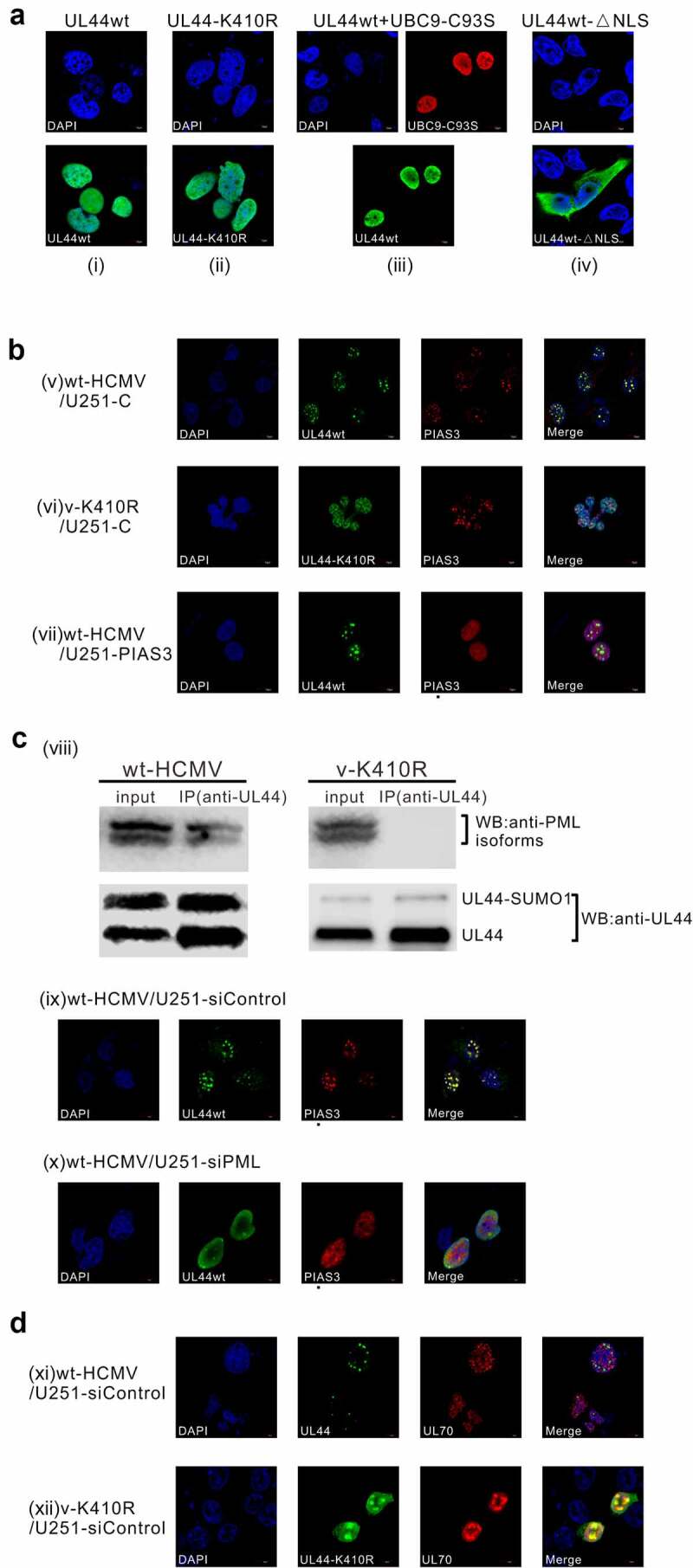
**SUMOylation at ^410^lysine of SCM site guides UL44 co-localization and interaction with ND10 structures during HCMV infection**. **(a)** Effect of K410 SUMOylation on UL44 nuclear localization in transfected cells. (i) pCMV-Myc-UL44 alone, (ii) pCMV-Myc-UL44-K410R alone, (iii) pCMV-Myc-UL44 together with pcDNA-HA-UBC9-C93S, or (iv) pCMV-Myc-UL44wt-ΔNLS alone was individually introduced into U251 cells. At 48 h post transfection, cells were fixed and permeabilized, followed by incubation with anti-UBC9/anti-Myc primary Abs and with Dylight 488/549-conjugated fluorescent secondary Abs. Through a confocal microscope, the subcellular localization of UL44 (WT or derivative forms, green) or UBC9-C93S (red) was visualized, with the cell nuclei stained by DAPI (blue). **(b)** Effect of K410 SUMOylation on UL44 subnuclear localization in HCMV infected cells. (v, vi) U251-C (control cells, carrying the empty pcDNA3.1(+) vector) or (vii) U251-PIAS3 cells (stably overexpressing PIAS3, carrying the pcDNA3.1-PIAS3 plasmid) were infected with wt-HCMV or v-K410R at an MOI of 1. At 48 hpi, fluorescent localization assays of UL44 (green) and PIAS3 (red) were performed with those infected cells as described above, using anti-UL44 or anti-PIAS3 as primary antibody. In a same field of view, “Merge” denoted the overlapping fluorescence from both the nuclei (blue) and expressed proteins. **(c)** The SCM-SUMOylation mediated UL44 protein co-localization and association with PML, the scaffold protein of ND10 subnuclear domains. (viii) Co-IP assays of UL44 with the cellular PML. U251 cells were infected with wt-HCMV or v-K410R at an MOI of 1, and 48 h later subjected to Co-IP assays using the Abs as indicated. (ix, x) Effect of endogenous PML interference on UL44 subnuclear localization in HCMV infected cells. U251-siControl (control cells, stably transduced using a control siRNA called siControl) or U251-siPML cells (stably transduced using siPML, a chemically synthesized siRNA that specially targets the CDS region of PML mRNA) were infected with wt-HCMV at an MOI of 1, followed by fluorescent localization assays of UL44 (green) and PIAS3 (red) at 48 hpi, using anti-UL44 and anti-PIAS3 as primary Abs, respectively. **(d)** The nuclear locations of UL44 and UL70 in HCMV infected cells. U251-siControl cells were infected with (xi) wt-HCMV or (xii) v-K410R at an MOI of 1, followed by fluorescent localization analyses of UL44 (green) and UL70 (red) at 48 hpi, using anti-UL44 and anti-UL70 as primary Abs, respectively. For each panel, the images were representative ones form three independent experiments

Since it has been reported that SUMOylation can regulate the subcellular localization of protein substrates and that UL44 needs transport into nucleus to execute its functions, we were urged to check whether the canonical SCM-specific SUMOylation might play any role in the localization property of UL44 into the nucleus. Indirect immunofluorescence localization assays showed that, when ectopically expressed in U251 cells, both the wild type and K410R mutant of UL44 proteins exhibited the same cellular distribution pattern in the nuclei (Figure 5a,b), and even competitive inhibition of the cellular SUMOylation machinery by co-expression with UBC9-C93S could not change the nuclear location pattern of UL44 (Figure 5a,c). In contrast, upon loss of the NLS motif, UL44wt-ΔNLS (wild type with truncation of NLS) turned to be located largely in the cytoplasm (Figure 5a,d). These observations suggested that the feature of UL44 localization in the nucleus is dependent on the C-terminal conserved NLS motif, but not affected by its canonical or non-canonical SUMO modifications.

Meanwhile, we investigated whether the physiological case of UL44 localization during HCMV infection might be influenced by the SCM-specific SUMOylation. As shown in virus-infected cells (Figure 5b, v and vi), UL44-wt proteins, as well as PIAS3, aggregated to appear as dot-like subnuclear structures, whereas the SCM-mutated UL44-K410R became more widely dispersed throughout the nucleus. This observation, considering previous report of PIAS3 to be localized partially with the PML nuclear bodies (PML-NBs) or namely ND10 subcompartments in COS-7 cells [55], hinted the possibility of UL44 localization being also at the subnuclear ND10 structures, owing to its possession of SCM-specific SUMOylation at K410. This hypothesis was further supported by the fact that wild-type UL44 encoded by wt-HCMV, but not the SCM-mutated UL44-K410R expressed by v-K410R, could co-localize with PML (representative component of ND10 structure) to display the same ND10-like punctate style of nuclear distribution (Fig S1). Of course, given the identified PIAS3–UL44 interaction, we also examined whether the nuclear co-localization of UL44 with PML was just due to the indirect recruitment by endogenous PIAS3. We thus constructed the U251-PIAS3 host cells capable of stably overexpressing PIAS3 for wt-HCMV infection (Fig S2). As seen in Figure 5b (v) versus (vii), over-expression of exogenous PIAS3 rendered itself a pronounced change in nuclear distribution from the punctate aggregation to more extensive dispersion; this, however, could not affect the UL44 nuclear distribution as clustered dot-like structures, thereby ruling out the indirect PIAS3 recruitment of UL44 to ND10 components.

Notably, we previously found the SUMOylation at K410 of UL44 could attenuate HCMV replication, and thus v-K410R, the mutated virus, owns a quicker viral DNA synthesis rate [35] (Fig S3, a). For this end, to exclude the possibility that UL44-wt and UL44-K410R distributions of nuclear foci and nuclear diffusion might, respectively, reflect viral pre-RC loci and the later viral DNA replication stage (Figure 5b; Fig S1), we also constructed U251-siUL54 host cells (Fig S2), in which no matter infected with wt-HCMV or v-K410R, the viral DNA synthesis was largely blocked by siUL54 (interfering in HCMV DNA polymerase) similarly (Fig S3, a). The immunofluorescence localization results, in good agreement with those observed in U251-C/U251-siControl host cells (Figure 5b, Fig S1/Fig S3), showed that UL44-wt of nuclear foci versus UL44-K410R of nuclear diffusion still kept unchanged when synchronizing both of the viral replications in stagnation; this difference in UL44 nuclear distribution, thereby not resultant from the inconsistent viral replicative progression, is caused by the K410R mutation of SCM site (Fig S3, b). Collectively, after HCMV infection, SCM-specific SUMOylation confers UL44 protein co-localization with the PML component of ND10 compartments, which is not the indirect recruitment by PIAS3.

Next, we sought to find what contributes to the co-localization of UL44 and PML during HCMV infection. Firstly, co-immunoprecipitation assays suggested that in virus-infected cells, UL44-wt could co-IP with the cellular PML whereas the SCM-mutated UL44-K410R could no longer form complexes with PML (Figure 5c, viii). Moreover, immunofluorescence localization analyses on the wt-HCMV infection showed that upon interference of endogenous PML by siPML, the UL44-wt distribution, as well as that of PIAS3, turned from the nuclear foci to nuclear extensive diffusion (Figure 5c, ix versus x). Therefore, the canonical SCM-specific SUMOylation allows UL44 protein to co-localize with ND10 structures through its association with the PML factor during HCMV infection.

### SCM SUMOylation-mediated UL44 localization to ND10 structure exerts a restricted effect on HCMV replication and reproduction

As revealed here and before [35], the PIAS3-directed K410 SUMOylation at UL44 SCM site can lead to (i) UL44 association and co-localization with PML factor of ND10 domains; (ii) attenuation of HCMV replication. Since we have ruled out (ii) as the antecedence of (i) (Fig S3), together with the consideration of ND10 role as part of the host cell antiviral immunity [29], it points to (i) as the causer of (ii) based on the available data and the results below.

To judge the UL44 distribution in context of viral RCs, immunofluorescence localization analyses were also assayed on UL44 and UL70 (HCMV primase, one of the core replication proteins in viral RCs). The result showed that UL44-wt, with SCM SUMOylation to locate at ND10 domains, could hardly co-localize with UL70, while UL44-K410R, deficient in SCM SUMOylation to be nuclear diffuse, shared a large area of localization overlap with UL70 and formed distinct viral replication regions that are beneficial for HCMV to replicate more efficiently (Figure 5d). Accordingly, it suggested that the SCM SUMOylation confines and gathers UL44 to ND10 subnuclear loci, which is adverse to the larger effective area as well as higher replicative efficiency of viral RCs and hence attenuates HCMV replication.

**Figure 6.**
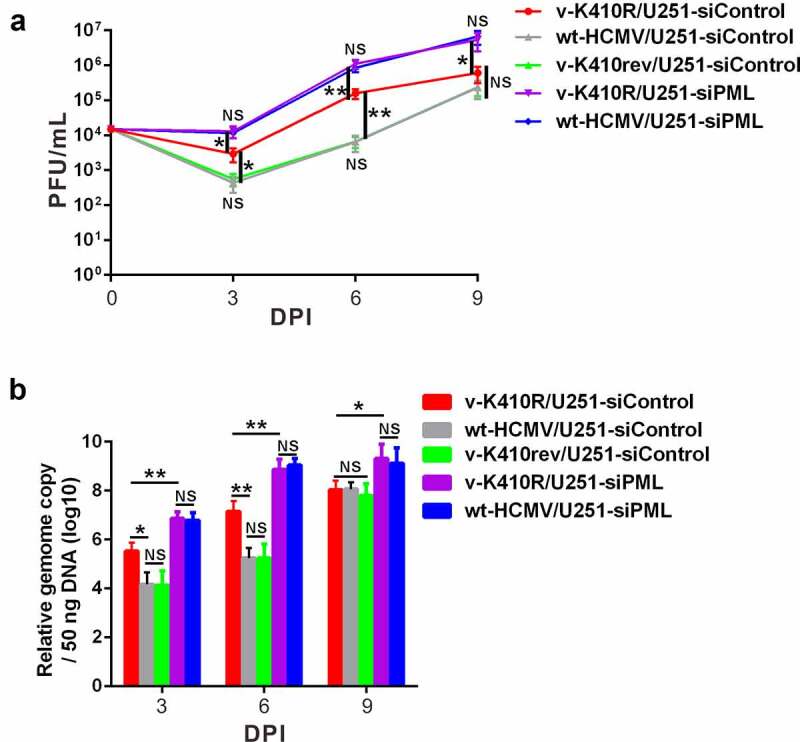
**SCM SUMOylation-mediated UL44 localization to ND10 structure restricts the replication and reproduction of HCMV**. U251-siControl cells and U251-siPML cells were individually infected with the indicated type of HCMV strain (wt-HCMV, v-K410R or v-K410rev) at an MOI of 0.5. At the indicated time points, **(a)** the viral titers and **(b)** HCMV DNA levels were determined by counting of the viral plaque numbers and by Real-Time PCR quantitation, respectively. The copies of viral genome were normalized to those of the cellular RNase P. In both (a) and (b), three repetitions were conducted for each set of assays in triplicate. Data represented the Average ± SD (standard deviation) from three repeated experiments. The Averages, between 2 groups or among more (≥3) groups, were appraised by usage of unpaired Student’s *t*-test or one-way ANOVA (analysis of variance) with Tukey’s *post hoc* test. NS denoted no significant difference. Differences were considered statistically significant when * denoted p < 0.05, ** denoted p < 0.01, and *** denoted p < 0.001

We finally monitored the viral progeny production of wild-type and mutated HCMVs at an MOI of 0.5 in U251 cells (Fig S2). As plotted in Figure 6a, in the siControl-transduced cells, v-K410rev (the revertant wild-type virus) exhibited a growth behavior similar to that of wt-HCMV, whereas the v-K410R mutant showed a stronger replication activity than wt-HCMV, reaching a maximal 1.5-log increased production of viral progeny at 6 days post infection (6 dpi). However, when disturbing ND10 formation by PML interference, the viral growth advantage of v-K410R over wt-HCMV became nearly eliminated in siPML-transduced cells, where both virus samples proliferated more quickly than in siControl-transduced cells. These data indicate a ND10-based restriction of HCMV replication, conferred partly by the SCM SUMOylation-mediated UL44 localization to ND10 structure and partly by other potential ways more than the UL44-ND10 co-localization.

Meanwhile, data of the viral genome copy determination agree well with those of the virus yield measurement above. The result similarly showed that in siControl-transduced cells, the genome of v-K410R mutant replicated more efficiently than that of wt-HCMV at 3 dpi and 6 dpi, and this genome replicative advantage turned to disappear at 9 dpi (Figure 6b). Again, in siPML-transduced cells, the genome replicative discrepancy between v-K410R and wt-HCMV was removed by PML interference that demolished the ND10 structures (Figure 6b).

Taken together, our results revealed that site-specific SUMOylation plays a critical role in the subnuclear localization of UL44, the HCMV polymerase processivity factor, to ND10 structures, which slows down the viral DNA replication to achieve the fine-tuned and self-controlled reproduction of herpesviruses during early infection.

## Discussion

Genomic DNA replication, highly conserved among all herpesviruses, is currently the target of most FDA-approved anti-herpes biochemical therapies [56]. Lytic replication of HCMV is an extremely complicated and organized process, including at least 11 identified HCMV-encoded proteins to fulfill its maximum DNA replication by now [3]. Our laboratory has long been committed to the extensive screening and investigation into the binary interactions between HCMV replication proteins and the host cell factors. For instance, Snapin, a key cytoplasmic vesicle-associated protein, was recently found to interact with either the HCMV primase UL70 or helicase UL105 using its different structural regions, therefore affecting the cellular distribution of the two core viral replication proteins [38,57]. DNAJ B6a and B6b, two proteins of the Hsp40/DNAJ family, could independently modulate the nuclear import and cytoplasmic accumulation of UL70, respectively [39]. During herpesviral infection, ND10 structures, a host cell antiviral subnuclear apparatus, depend on SUMOylation of their components to fulfill own assembly and functionality in restriction of the viral DNA replication. In this study, to unveil the role of SUMO modification on the viral polymerase processivity factor UL44 in HCMV replication, we focused on screening UL44-interacting proteins involved in the cellular SUMOylation pathway, the ones besides the previously identified SUMO-conjugating enzyme UBC9. We then identified a direct protein interaction between UL44 and PIAS3, an annotated E3 SUMO ligase, revealed to promote site-specific SUMOylation of UL44 at its conserved ^410^lysine residue within the single canonical SUMO Conjugation Motif (SCM, i.e., ψKxE). This SCM-specific SUMOylation targets UL44 to ND10 subnuclear structures, resulting in restriction on HCMV replication.

The process of SUMOylation is analogous to that of ubiquitination, both PTMs catalyzed by a three-step enzymatic cascade: the 11-kDa SUMO moiety is first activated by the E1 SUMO-activating enzyme in an ATP-dependent manner, subsequently transferred to the E2 SUMO-conjugating enzyme (UBC9), and finally attached to the ε-amino group of a target lysine within the protein substrate either still by UBC9 or by another alternative E3 SUMO ligase [14]. Currently, there are already several E2 ubiquitin-conjugating enzymes and over 600 E3 ubiquitin ligases discovered in eukaryotes, but only one single E2 SUMO-conjugating enzyme (UBC9) and a limited number of E3 SUMO ligases have been identified [58]. Particularly, PIAS (protein inhibitors of activated STAT, such as PIAS-1/3/xα/xβ) proteins are a representative family of E3-type SUMO-1 ligases, capable of binding SUMO-1 and UBC9 to SUMOylate themselves and their interacting partners like various transcriptional factors [59]. Considering the PIAS3 role in inhibition of activated STAT3 pathway [46,47] and the identified PIAS3 interaction with UL44, there might be a possibility that the STAT3 activity could be enhanced by competitive UL44 binding to PIAS3 during HCMV infection. Members of the PIAS family, as E3 SUMO ligases, have been reported to improve the efficiency of SUMOylation, to display certain protein substrate specificity for SUMO conjugation, or to interact with other SUMOylated proteins [59]. All these properties of PIAS family are dependent on their central conserved SP-RING finger domain. Previously, the HCMV DNA polymerase processivity factor, UL44, was revealed to be bound and SUMOylated by UBC9 [23]. Here, we characterized an E3 SUMO ligase activity of PIAS3 toward UL44 both *in vivo* and *in vitro*, which renders the UL44 SUMOylation system more integral. PIAS3 is able to enhance the UBC9-based SUMO modifications on UL44, using SUMO-1, 2, or 3 peptides. This SUMOylation-promoting activity of PIAS3 can be inactivated by the C334S mutation of SP-RING domain, or by siPIAS3 interference during infection. In the SUMO-modifying *in vitro* assays, C334S mutation of PIAS3 led to a whole malfunction of the human SUMOylation machinery; this might be due to the formation of inactive ternary UL44-PIAS3_C334S_-UBC9 complex that interrupts the normal catalysis for SUMO conjugation. Of note, we recently found among the multiple sites modified by UBC9, ^410^lysine within SCM of UL44 is the protein’s major SUMOylation site, yet leaving the selective mechanism unknown [35]. Interestingly, PIAS3 was discovered here to be just responsible for this K410 SUMO-linking preference of UL44.

SUMOylation plays a pleiotropic role in protein functions of host cells and infecting viruses, especially in regulation of protein–protein interactions as well as in direction of the SUMO-attached proteins to certain subcellular or subnuclear localization [14]. One representative example can be observed in the SUMOylation dependency of ND10 assembly and functionality. ND10 subnuclear domains act in host cells not only as antiviral devices made from SUMOylated proteins, but also as hot spots for SUMOylation reaction bearing SUMO peptides and most of the cellular SUMO-modifying enzymes [33]. In particular, even the large majority of ND10-interacting proteins are also SUMO-linked [33]. For instance, during HAdV (human adenovirus) infection, the SCM site-specific SUMOylation of E2A protein in viral RCs (replication compartments) was found to mediate the RC-ND10 proximity and interaction [30]. In our study, the SCM-specific SUMOylation of UL44 during infection, likewise, guides the UL44-ND10 co-localization and association, forming the UL44 distribution of nuclear foci that restricts the larger area of effective viral RCs and accordingly hampers higher HCMV replicative efficiency. This ND10-based restriction on HCMV replication keeps consistent with the ND10 role as host cell antiviral apparatus. In contrast, v-K410R encodes UL44-K410R that lacks the SCM-specific SUMOylation to exhibit the nuclear distribution of wide diffusion, and instead, the mutant virus replicates more efficiently and quickly upon the relief of ND10 restriction to UL44 intranuclear localization.

Our results are reminiscent of several previous observations on UL44 nuclear localizations. Both Sinigalia *et al*. and Scherer *et al*. from two teams once showed that overexpression of SUMO-1/3 in HCMV-infected cells would contribute to alteration of UL44 nuclear distribution to a more diffuse pattern and meanwhile cause a positive effect on HCMV replication [43,60]; this might be partially explained according to our work that overexpression of SUMO, for some unknown reasons, disabled the original UL44 localization in ND10 domains to become more widely dispersed throughout the nucleus, thereby relieving HCMV replication from the ND10 antiviral defense of host cells and correspondingly promoting viral progeny production, a case similar to that observed in the v-K410R infected cells. Moreover, as stated above, formation of the viral DNA RCs takes place in proximity to ND10 domains upon herpesvirus infection. Reminiscently, Strang *et al*. discovered that during the progression of HCMV infection, viral DNA synthesis occurs at the periphery of RCs where the vast majority of UL44 population concentrates to form a surrounding layer, and then, the replicated viral DNA localizes into the interior of RCs where a small amount of UL44 proteins also exist to be speculated functional for late viral gene transcription and viral genome package [61]. Agreeing well with our findings, this observation is understandable and interpretable: the HCMV polymerase processivity factor UL44 was first confined and tethered by ND10 domains to concentrate at the periphery of RCs; then, with the virus counteraction against host immunity and the final collapse of ND10 structures, UL44 was released from the ND10 restriction to step into the interior of RCs for late viral gene transcription and viral package, thus propelling the progression of viral proliferation.

In addition, the presented multiple sequence alignment suggested that HCMV UL44 and other CMV homologues share a great sequence similarity mainly in their N-terminal part carrying two core subdomains, while the C-terminal portion covers most of the non-homologous zone in these proteins. However, the last 29 amino acids at the end of C-terminus constitute a highly homologous region, especially in possession of the NLS sequence accompanied by the ψ^410^KxE SUMOylation motif. As revealed here, the conserved K410 residue within this single canonical SCM of UL44, just the PIAS3-regulated UL44 major SUMOylation site, is of great importance for UL44 function implicated in HCMV replication. Mutation of ^410^lysine generated a series of successive effects during infection: it resulted in a remarkable decline in the UL44 SUMOylation level, disabled the U44 association and co-localization with PML, changed the UL44 nuclear distribution from ND10 foci to widespread diffusion, and more importantly, relieved HCMV from the ND10 restriction of host cells on viral replication and growth. To sum up, the site-specific SUMOylation can mediate the HCMV replication protein localization to ND10 structures as well as the ensuing inhibition of viral replication, representing a novel regulatory mechanism for the herpesviral reproduction. In fact, HCMV exhibits slower replication cycle than other herpesviruses; too fast viral growth will soon lyse and ruin the host cells where viruses survive. Thus, from the view of viral evolution, the stringent sequence conservation of that single canonical ψKxE SUMOylation site, genetically retained in HCMV polymerase processivity factor to exert an ND10 restriction of viral own replication, might guarantee the virus a fine-tuned and self-controlled slower replicative progression. This is harmonious for virus-host coexistence during early infection as well as beneficial for viral long-term parasitism and reproduction.

## Supplementary Material

Supplemental Material

## Data Availability

The authors confirm that the data supporting the findings of this study are available within the article and its supplementary materials. Raw data are available from the corresponding author JC on request.
