# Supplementary figures and images for: Site-specific SUMOylation of viral polymerase processivity factor: a way of localizingtoND10 subnuclear domains for restricted and self-controlled reproduction of herpesvirus
Source: Virulence. 2021 Dec 16;12(1):2883–901. doi: 10.1080/21505594.2021.2000689 (PMC8923073; doi:10.1080/21505594.2021.2000689)

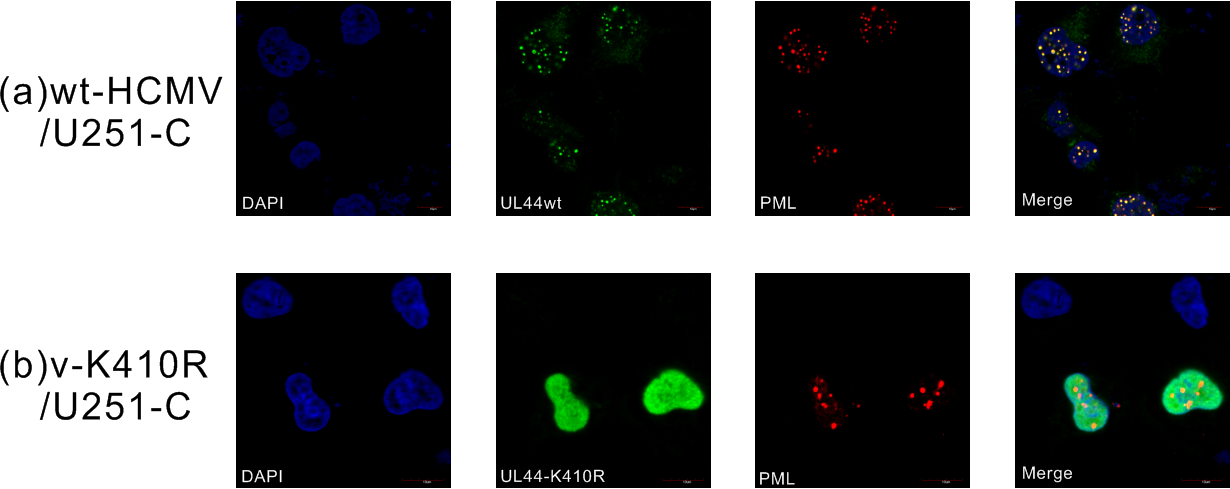

Supplement: Supplemental Material [file KVIR_A_2000689_SM3326.zip › supplementary/Figure S1.tif]

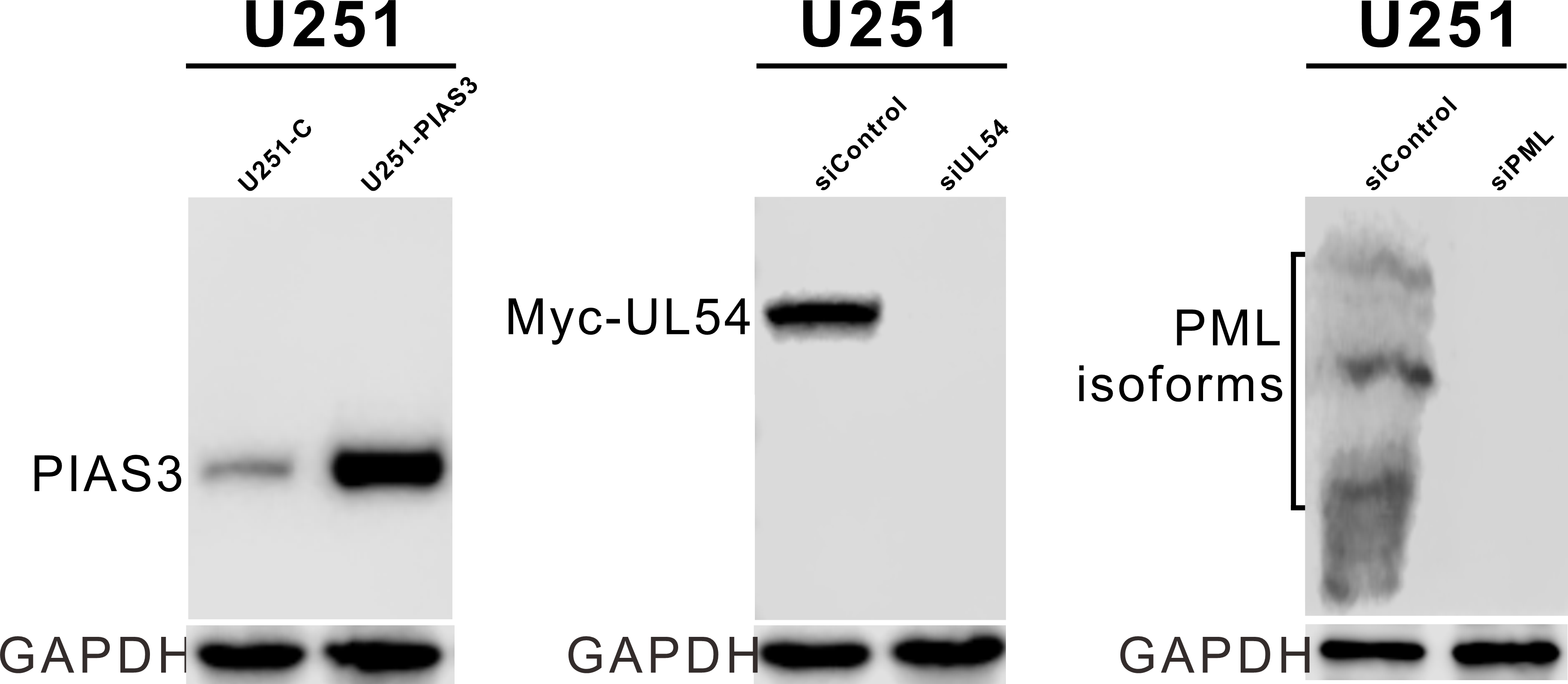

Supplement: Supplemental Material [file KVIR_A_2000689_SM3326.zip › supplementary/Figure S2.tif]

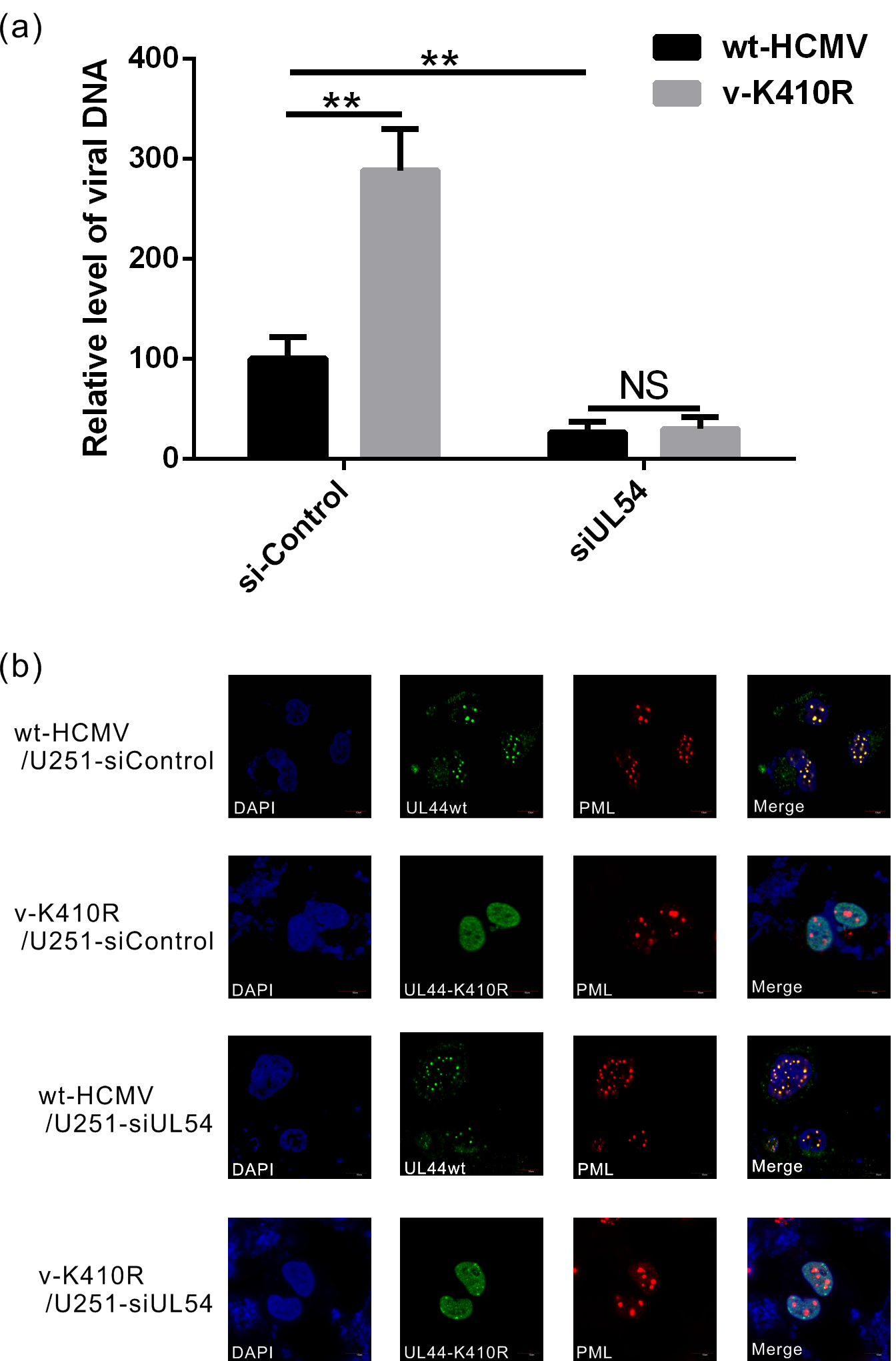

Supplement: Supplemental Material [file KVIR_A_2000689_SM3326.zip › supplementary/Figure S3.tif]
